# Cinnamon extract inhibits α-glucosidase activity and dampens postprandial glucose excursion in diabetic rats

**DOI:** 10.1186/1743-7075-8-46

**Published:** 2011-06-29

**Authors:** H Mohamed Sham Shihabudeen, D Hansi Priscilla, Kavitha Thirumurugan

**Affiliations:** 1 Structural Biology Lab, Centre for Biomedical Research, SBST, VIT University, Vellore, India

## Abstract

**Background:**

α-glucosidase inhibitors regulate postprandial hyperglycemia (PPHG) by impeding the rate of carbohydrate digestion in the small intestine and thereby hampering the diet associated acute glucose excursion. PPHG is a major risk factor for diabetic vascular complications leading to disabilities and mortality in diabetics. *Cinnamomum zeylanicum*, a spice, has been used in traditional medicine for treating diabetes. In this study we have evaluated the α-glucosidase inhibitory potential of cinnamon extract to control postprandial blood glucose level in maltose, sucrose loaded STZ induced diabetic rats.

**Methods:**

The methanol extract of cinnamon bark was prepared by Soxhlet extraction. Phytochemical analysis was performed to find the major class of compounds present in the extract. The inhibitory effect of cinnamon extract on yeast α-glucosidase and rat-intestinal α-glucosidase was determined *in vitro *and the kinetics of enzyme inhibition was studied. Dialysis experiment was performed to find the nature of the inhibition. Normal male Albino wistar rats and STZ induced diabetic rats were treated with cinnamon extract to find the effect of cinnamon on postprandial hyperglycemia after carbohydrate loading.

**Results:**

Phytochemical analysis of the methanol extract displayed the presence of tannins, flavonoids, glycosides, terpenoids, coumarins and anthraquinones. *In vitro *studies had indicated dose-dependent inhibitory activity of cinnamon extract against yeast α-glucosidase with the IC _50 _value of 5.83 μg/ml and mammalian α-glucosidase with IC _50 _value of 670 μg/ml. Enzyme kinetics data fit to LB plot pointed out competitive mode of inhibition and the membrane dialysis experiment revealed reversible nature of inhibition. *In vivo *animal experiments are indicative of ameliorated postprandial hyperglycemia as the oral intake of the cinnamon extract (300 mg/kg body wt.) significantly dampened the postprandial hyperglycemia by 78.2% and 52.0% in maltose and sucrose loaded STZ induced diabetic rats respectively, compared to the control. On the other hand, in rats that received glucose and cinnamon extract, postprandial hyperglycemia was not effectively suppressed, which indicates that the observed postprandial glycemic amelioration is majorly due to α-glucosidase inhibition.

**Conclusions:**

The current study demonstrates one of the mechanisms in which cinnamon bark extract effectively inhibits α-glucosidase leading to suppression of postprandial hyperglycemia in STZ induced diabetic rats loaded with maltose, sucrose. This bark extract shows competitive, reversible inhibition on α-glucosidase enzyme. Cinnamon extract could be used as a potential nutraceutical agent for treating postprandial hyperglycemia. In future, specific inhibitor has to be isolated from the crude extract, characterized and therapeutically exploited.

## Background

In individuals with type 2 diabetes, nutrient intake related first-phase insulin response is severely diminished or absent resulting in persistently elevated postprandial glucose (PPG) throughout most of the day [1]. This is due to the delayed peak insulin levels which are insufficient to control PPG excursions adequately [2]. Postprandial hyperglycemia is a major risk factor for micro- and macro vascular complications associated with diabetes [3,4] and so controlling postprandial plasma glucose level is critical in the early treatment of diabetes mellitus and in reducing chronic vascular complications [5]. The acute glucose fluctuations during the postprandial period exhibits a more specific triggering effect on oxidative stress than chronic sustained hyperglycemia which suggests that the therapy in type 2 diabetes should target not only hemoglobin A1c and mean glucose concentrations but also acute glucose swings [6,7].

Mammalian α-glucosidase anchored in the mucosal brush border of the small intestine catalyzes the end step digestion of starch and sucrose that are abundant carbohydrates in human diet [8]. α-glucosidase inhibitors (AGI) delay the breakdown of carbohydrate in small intestine and diminish the postprandial blood glucose excursion in diabetic subjects [9,10] and thus have a lowering effect on postprandial blood glucose and insulin levels. Commercially available α-glucosidase inhibitors such as acarbose, miglitol and voglibose are widely used to treat patients with type 2 diabetes [11,12]. AGI is shown to reduce the insulin requirements for type 1 diabetes and it also improves reactive hypoglycemia [10]. As the α-glucosidase inhibitors exhibit therapeutic effect by restricting carbohydrate absorption, the undigested carbohydrate dislodged to the colon undergoes fermentation by colonic flora to result in adverse effects such as flatulence, abdominal discomfort and diarrhoea [13]. However the adverse effects are dose dependent and get reduced with the duration of therapy [14,15].

Several α-glucosidase inhibitors have been isolated from medicinal plants to develop as an alternative drug with increased potency and lesser adverse effects than the existing drugs [16]. Cinnamon is used in traditional medicine for treating diabetes and it was found to have insulin secretagogue property [17] and insulin sensitizing property [18]. Besides the antidiabetic effect, the cinnamon bark and cinnamon oil have been reported to possess antioxidant activity [19], antinociceptive property [20], acaricidal property [21], and activity against urinary tract infections [22]. In a human clinical trial, it was found that intake of cinnamon with rice pudding reduced postprandial blood glucose and delayed gastric emptying [23].

Ahmad Gholamhoseinian [24] screened 200 Iranian medicinal plants *in vitro *and reported that the cinnamon extract exhibited strong inhibition on yeast α-glucosidase. However, the nature of the enzyme inhibition was not studied in detail. As most of the plant derived inhibitors showing effective inhibition on yeast α-glucosidase do not effectively inhibit the mammalian α-glucosidase, we have prompted to evaluate the same. In addition, we have studied the effect of cinnamon extract on postprandial glucose excursion associated with disaccharides and monosaccharide challenge in normal and STZ induced diabetic rats.

## Methods

### Plant materials

*Cinnamomum zeylanicum *(CZ) bark was collected form Mailadumpara, Kerala and authenticated by Angelin Vijayakumari, Head, Department of Plant Biology and Biotechnology, Voorhees College, Vellore, India. A voucher specimen of the plant (ID: VRC001) was deposited in the Herbarium Center, Voorhees College, Vellore, India.

### Extraction methods

Shade dried bark (50 g) was milled and extracted using methanol (250 ml) in Soxhlet apparatus for 8 hours. Then, the extract was evaporated to dryness and the final dry chocolate colour crude extract was stored in dark at -20°C until used for the experiments.

### Phytochemical analysis

The phytochemical analysis of cinnamon bark extract has been performed to find the presence of major secondary metabolites like flavonoids, tannins, saponins, steroid, glycosides, coumarins, anthraquinones and alkaloids. Standard protocols according to Trease and Evans [25] and Harborne [26] were followed to analyze tannins, flavonoids, glycosides, terpenoids, alkaloids, coumarins, and anthraquinones. Steroidal rings analysis was performed following method described by Sofowora [27]. Saponins were analysed by following the protocol described by Wall [28].

### Enzyme assay

*p*-Nitrophenyl-α-D-glucopyranoside (PNPG), Yeast α-glucosidase (EC 3.2.1.20), sodium phosphate salts and sodium carbonate were purchased from Sisco (SRL), India. Rat-intestinal acetone powder was obtained from Sigma (USA). Acarbose was bought from Bayer pharmaceuticals, India. α- glucosidase inhibitory activity was performed following the modified method of Pistia Brueggeman and Hollingsworth [29,30]. Mammalian α- glucosidase was prepared following the modified method of Jo [31]. Rat-intestinal acetone powder (200 mg) was dissolved in 4 ml of 50 mM ice cold phosphate buffer and sonicated for 15 minutes at 4°C. After vigorous vortexing for 20 minutes, the suspension was centrifuged (10,000 g, 4°C, 30 minutes) and the resulting supernatant was used for the assay. A reaction mixture containing 50 μl of phosphate buffer (50 mM; pH 6.8), 10 μl of yeast or Rat α-glucosidase (1 U/ml) and 20 μl of plant extract of varying concentrations was pre-incubated for 5 min at 37°C, and then 20 μl of 1 mM PNPG was added to the mixture as a substrate. After further incubation at 37°C for 30 min, the reaction was stopped by adding 50 μl of Na_2_CO_3 _(0.1 M). All the enzyme, inhibitor and substrate solutions were made using the same buffer. Acarbose was used as a positive control and water as negative control. Enzymatic activity was quantified by measuring the absorbance at 405 nm in a microtiter plate reader (Bio-TEK, USA). Experiments were done in triplicates. The percentage of enzyme inhibition by the sample was calculated by the following formula: % Inhibition = {[(AC - AS)/AC] ×100}, where AC is the absorbance of the control and AS is the absorbance of the tested sample. The concentration of an inhibitor required to inhibit 50% of enzyme activity under the mentioned assay conditions is defined as the IC_50 _value.

### Kinetics of α-glucosidase inhibition by CZ

The mode of inhibition of CZ extract against mammalian α-glucosidase activity was measured with increasing concentrations of PNPG (0.5,1,2 and 4 mM) as a substrate in the absence and presence of CZ at 0.5 mg/ml and 1 mg/ml. Optimal amounts of CZ used were determined based on the enzyme inhibitory activity assay. Mode of inhibition of CZ was determined by Lineweaver-Burk plot analysis of the data calculated following Michaelis-Menten kinetics [32,33].

### Dialysis for reversibility of CZ action

α-glucosidase (100 U/ml) was incubated with CZ (23.5 mg/ml) in 0.5 ml of sodium phosphate buffer (50 mM, pH 6.7) for 2 h at 37°C and dialyzed against sodium phosphate buffer (5 mM, pH 6.7) at 4°C for 24 h, changing the buffer every 12 h. Another premixed-enzyme solution (0.5 ml) was kept at 4°C for 24 h without dialysis for the control experiment. Reversibility of CZ has been determined by comparing the residual enzyme activity after dialysis with that of non-dialyzed one [34,35].

### Experimental animals

Adult male Albino wistar rats were maintained during the experiments in the animal house, Center for Biomedical Research, VIT University, Vellore. 12-13 weeks old rats, weighing 160-210 g were kept in polycarbonate cage housed in a room with a 12-h light/12-h dark cycle at 25 ± 2°C, fed with standard rodent diet and water ad libitum. All animal procedures were approved by the ethical committee in accordance with our institutional Animal Ethics Committee, 1333/C/10/CPCSEA.

### Induction of diabetes

Rats previously fasted for 16 h were given single intraperitoneal injection of 45 mg/kg body wt. streptozotocin (Sigma, USA) dissolved in freshly prepared citrate buffer (0.1 M, pH4.5). Animals with fasting blood glucose over 250 mg/dl, three days after streptozotocin administration were considered diabetic and they received treatment similar to that of normal rats.

### Maltose and sucrose loading in normal rats

Total of eighteen rats were segregated into three groups of six animals each. After 16 hours fasting, Group 1 had received maltose or sucrose (2 g/kg body wt; p.o.) as the diabetic control. Group 2 was coadministered with maltose or sucrose (2 g/kg body wt; p.o.) and CZ extract (300 mg/kg body wt; p.o.). Group 3 was coadministered with maltose or sucrose (2 g/kg body wt; p.o.) and acarbose (5 mg/kg body wt; p.o.). Selected dosages of cinnamon extract and acarbose were determined to be safe based on the previous studies [36-38]. Blood glucose level was measured before and 30, 60 and 120 minutes after the maltose or sucrose loading using a Glucometer (One touch Horizon™). The change in blood glucose from the basal level after the carbohydrate load was analysed and represented as delta blood glucose.

### Maltose and sucrose loading in diabetic rats

Total of 24 rats were sorted into four groups of six animals each. After 16 hours fasting, they were given single intraperitoneal injection of 45 mg/kg body wt. streptozotocin (Sigma, USA). Group 1 had received maltose or sucrose (2 g/kg body wt; p.o.) as the diabetic control. Group 2 was coadministered with maltose or sucrose (2 g/kg body wt; p.o.) and CZ extract (300 mg/kg body wt; p.o.). Group 3 was coadministered with maltose or sucrose (2 g/kg body wt; p.o.) and CZ extract (600 mg/kg body wt; p.o.); Group 4 was coadministered with maltose or sucrose (2 g/kg body wt; p.o.) and acarbose (5 mg/kg body wt; p.o.). Blood glucose level was measured at 0, 30, 60, and 120 minutes after the maltose or sucrose loading using a Glucometer (One touch Horizon™). Deviation in blood glucose concentration from the basal value was analysed and represented as delta blood glucose.

### Glucose loading in normal rats

Total of twelve normal rats were segregated into two groups of six animals each. After 16 hours fasting, Group 1 had received glucose (2 g/kg body wt; p.o.) as the control. Group 2 was coadministered with glucose (2 g/kg body wt; p.o.) and CZ extract (300 mg/kg body wt; p.o.). Blood glucose level was measured before and 30, 60 and 120 minutes after the glucose loading using a Glucometer (One touch Horizon™). The change in blood glucose from the basal level after the oral load was analysed and represented as delta blood glucose.

### Glucose loading in diabetic rats

Total of twelve diabetic rats were segregated into two groups of six animals each. After 16 hours fasting, Group 1 had received glucose (2 g/kg body wt; p.o.) as the control. Group 2 was coadministered with glucose (2 g/kg body wt; p.o.) and CZ extract (300 mg/kg body wt; p.o.). Blood glucose level was measured before and 30, 60 and 120 minutes after the glucose loading using a Glucometer (One touch Horizon™). The change in blood glucose from the basal level after the oral load was analysed and represented as delta blood glucose.

### Statistical analyses

Statistical analysis was performed using t-test or one-way analysis of variance (ANOVA) followed by Dunnett's Multiple Comparison Test using GraphPad Prism software. P-values of less than 0.05 were considered to be statistically significant. The delta blood glucose levels were expressed as mean ± SE for six animals in each group.

## Results

### Phytochemical constituents of CZ

Phytochemical analysis of the cinnamon extract indicated the presence of flavonoids, glycosides, coumarins, alkaloids, anthraquinone, steroids, tannins and terpenoids.

### *In vitro *α-glucosidase inhibition by CZ

**Figure 1:**
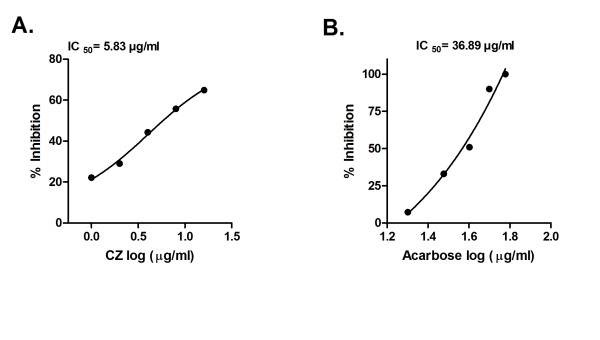
**Inhibition of yeast α-glucosidase by CZ extract**. A. Inhibition of α-glucosidase by CZ extract at various concentrations (1-16 μg/ml). B. Inhibition α-glucosidase by acarbose at various concentrations (1-60 μg/ml). The α-glucosidase inhibition was analyzed by measuring *p*-nitrophenol released from PNPG at 405 nm after 30 minutes of incubation at 37°C. Results are expressed as mean of percent inhibition ± S.E.M against log 10 concentration of inhibitor.

**Figure 2:**
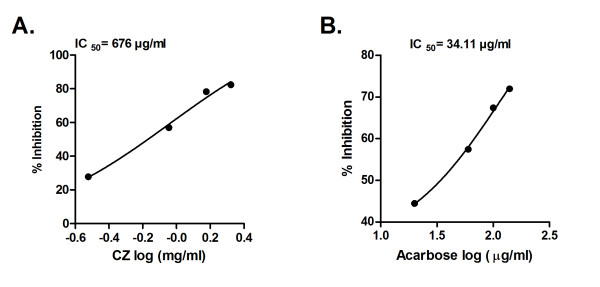
**Inhibition of Mammalian α-glucosidase by CZ extract**. A. Inhibition of mammalian α-glucosidase by CZ extract at various concentrations (0.3-2.1 mg/ml). B. Inhibition mammalian α-glucosidase by acarbose at various concentrations (20-140 μg/ml). The α-glucosidase inhibition was analyzed by measuring *p*-nitrophenol released from *p*NPG at 405 nm after 30 minutes of incubation at 37°C. Results are expressed as mean of percent inhibition ± S.E.M against log 10 concentration of inhibitor.

Yeast α-glucosidase inhibition potential of the CZ extract and acarbose was measured (Figure 1A). It displays effective inhibition of α-glucosidase by CZ extract with IC _50 _value of 5.83 μg/ml. Acarbose used as the positive control showed IC _50 _value of 36.89 μg/ml (Figure 1B), under similar assay conditions. CZ extract and acarbose inhibited rat-intestinal α-glucosidase with IC _50 _value of 676 μg/ml and 34.11 μg/ml, respectively (Figure 2A and 2B).

### Mode of α-glucosidase inhibition by CZ

**Figure 3:**
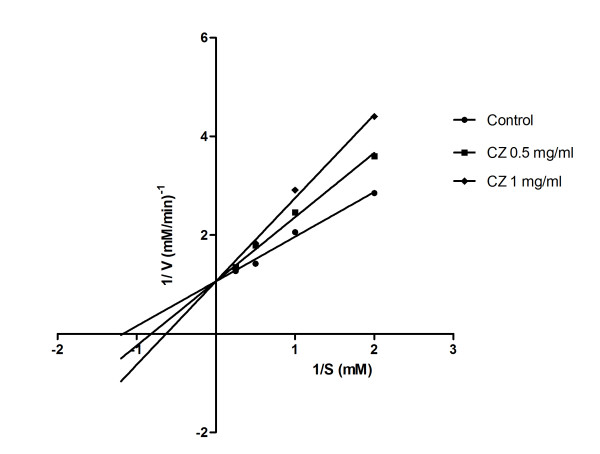
**Mode of α-glucosidase inhibition by CZ extract**. Lineweaver-Burk plot of α- glucosidase inhibition by CZ. α-glucosidase was treated with various concentrations of *p*NP-glycoside (0.5-4 mM) in the absence or presence of CZ at two different concentrations (0.5 and 1 mg/ml). The kinetics assay has been performed after incubating the mixture at 37°C for 30 min.

The mode of inhibition of CZ extract on rat-intestinal α-glucosidase activity was analyzed using LB plot. The double-reciprocal plot displayed competitive inhibition of the enzyme activity (Figure 3). The K_m _value increased with increase in the CZ concentration and V_max _remained unaltered (Table 1).

**Table 1:** Kinetic analysis of α-glucosidase inhibition by CZ

CZ (mg/ml)	V_max _(mM/min)	K_m _(mM)
0	0.94	0.85

0.5	0.94	1.22

1	0.94	1.59

Table 1. α-glucosidase with different concentrations of PNPG (0.5-4 mM) was incubated in the absence and presence of CZ at two different concentrations (0.5 and 1 mg/ml) at 37°C for 30 min. K_m _and V_max _were calculated from Lineweaver-Burk plot.

### Reversibility of CZ action

**Figure 4:**
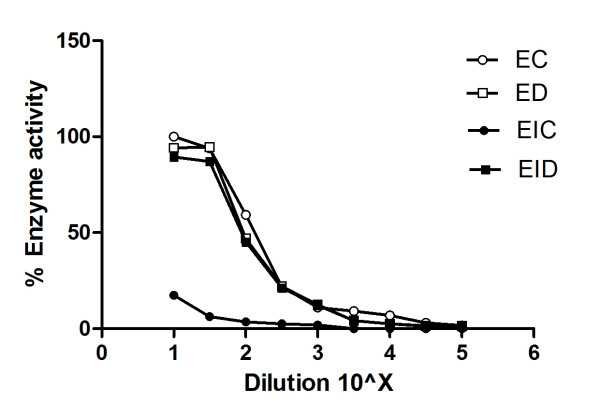
**Reversibility of CZ action**. α-glucosidase (100 U) was incubated with CZ (23.5 mg) in 0.5 ml of sodium phosphate buffer (50 mM; pH 6.7) for 2 h at 37°C and dialyzed against sodium phosphate buffer (5 mM; pH 6.7) at 4°C for 24 h. Reversibility of CZ was determined by comparing the residual enzyme activity after dialysis with that of non-dialyzed one. α-glucosidase alone (EC, ED) and the complex of α-glucosidase and CZ (EIC, EID) were dialyzed against 5 mM sodium phosphate buffer (pH 6.7) at 4°C (ED, EID) or were kept at 4°C (EC, EIC) for 24 h.

The enzyme activity of α-glucosidase was almost completely recovered after the dialysis, shown by the enzyme mixed inhibitor curve (EID) that was similar to the curves of enzyme control without dialysis (EC) and with dialysis (ED) (Figure 4). Proximal running of ED as experimental control along with EC and EID ensures that dialysis alone does not greatly affect the enzyme activity. However, the non-dialyzed mixture of enzyme and extract (EIC) showed its inhibited activity.

### Maltose loading in normal rats

**Figure 5:**
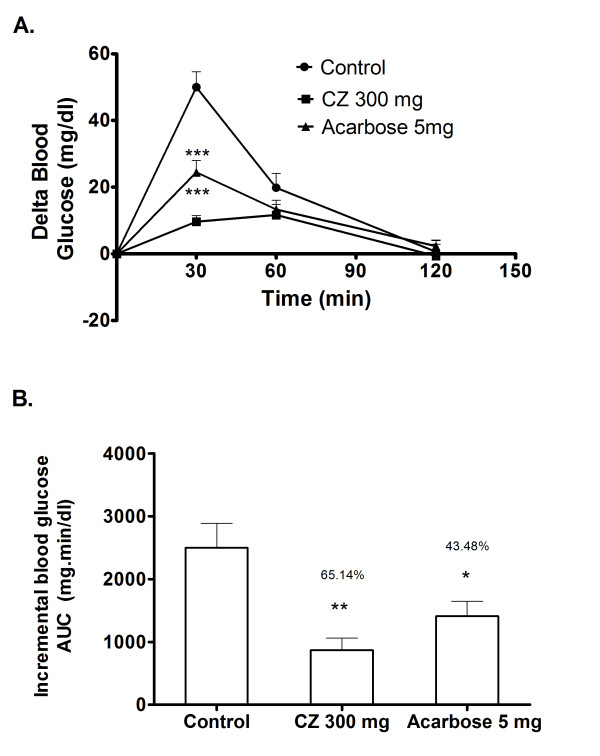
**Inhibitory effects of CZ on blood glucose after maltose loading in normal rats**. The normal rats fasted for 16 h received maltose (2 g/kg body wt; p.o.) and dose of CZ (300 mg/kg body wt; p.o.) by gastric intubation. Control group received maltose (2 g/kg body wt; p.o.) alone, and the drug control group received maltose (2 g/kg body wt; p.o.) plus acarbose (5 mg/kg). Blood glucose was measured at 0, 30, 60 and 120 min after food administration. A. The glycemic response curve in normal rats after maltose challenge. B. The incremental AUC_0-120 min _in normal rats after maltose administration. Data are expressed as the mean ± S.E, n = 6. *, P < 0.05 vs. control; **, P < 0.01 vs. control; ***, P < 0.001 vs. control.

Postprandial blood glucose variation was measured after loading maltose to the normal rats with and without the coadministration of CZ extract. In the control group, blood glucose level increased by an average of 50 mg/dl at 30 minutes after the maltose load. In the group that received CZ extract along with maltose, the 30 minutes post-load glucose level increased only marginally by 9 mg/dl on an average (Figure 5A). This indicates the potency of CZ extract to significantly suppress high maltose diet associated postprandial glucose elevation. Compared to control, the whole glycemic response is reduced by 65.1% on CZ treatment (Figure 5B).

### Maltose loading in diabetic rats

**Figure 6:**
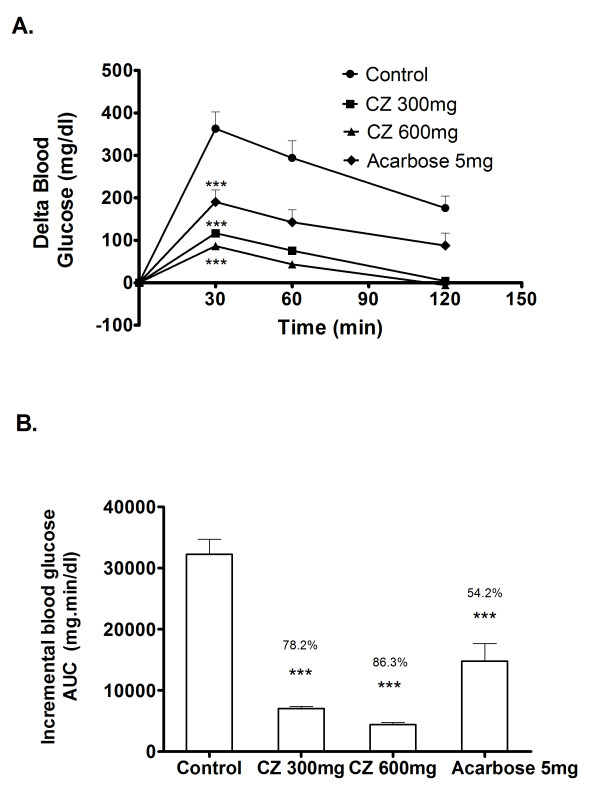
**Inhibitory effect of CZ on blood glucose after maltose loading in diabetic rats**. The diabetic rats fasted for 16 h received maltose (2 g/kg body wt; p.o.) and different doses of CZ (300 mg/kg body wt; p.o. and 600 mg/kg body wt; p.o.) by gastric intubations. Control animals were given only maltose (2 g/kg body wt; p.o.) and the drug control group received maltose (2 g/kg body wt; p.o.) plus acarbose (5 mg/kg). Blood glucose was monitored at 0, 30, 60 and 120 min after food administration. The result shows the significantly impeded 30 minutes post-load glucose level in the CZ 300 mg and CZ 600 mg treated group compared to control. Data are expressed as the mean ± S.E, n = 6. *, P < 0.05 vs. control; **, P < 0.01 vs. control; ***, P < 0.001 vs. control.

As CZ extract exhibited appreciable postprandial blood glucose lowering effect in the normal rats, we examined its inhibitory effect on STZ induced diabetic rats. In the control group, blood glucose level increased to an average of 362 mg/dl above the basal level 30 min after CZ administration and decreased thereafter (Figure 6A). However, the rise of the post-load blood glucose has been significantly impeded in a dose dependent fashion on coadministering CZ with maltose at different doses (300, 600 mg/kg body wt.). Similar kind of suppression effect was observed in the group that received acarbose (5 mg/kg body wt.) as the positive control along with maltose. Compared to control, the whole glycemic response is reduced by 78.2%, 86.3% and 54.2% when treated with 300, 600 mg/kg body wt. of CZ and 5 mg/kg body wt. of acarbose, respectively (Figure 6B).

### Sucrose loading in normal rats

**Figure 7:**
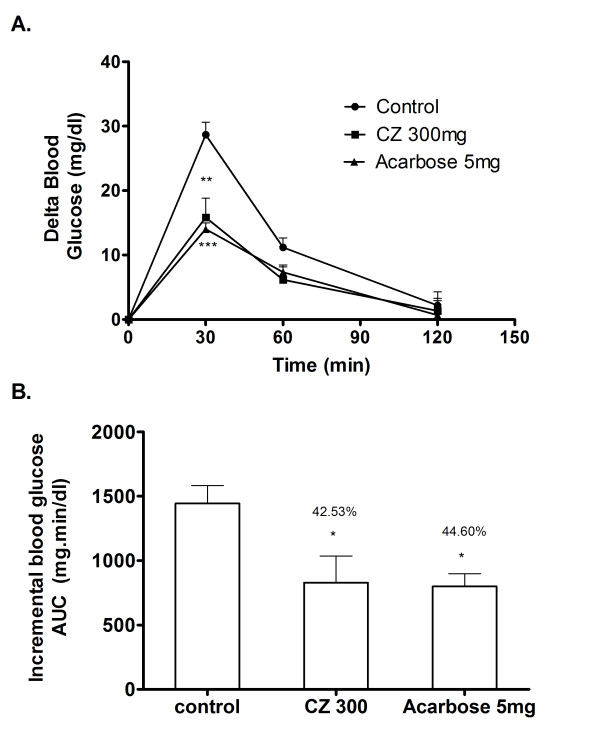
**Inhibitory effects of CZ on blood glucose after sucrose loading in normal rats**. The normal rats fasted for 16 h received sucrose (2 g/kg body wt; p.o.) and dose of CZ (300 mg/kg body wt; p.o.) by gastric intubation. Control group received sucrose (2 g/kg body wt; p.o.) alone and the drug control group received sucrose (2 g/kg body wt; p.o.) plus acarbose (5 mg/kg). Blood glucose was measured at 0, 30, 60 and 120 min after food administration. A. The glycemic response curve in normal rats after sucrose challenge. B. The incremental AUC_0-120 min _in normal rats after sucrose administration. Data are expressed as the mean ± S.E, n = 6. *, P < 0.05 vs. control; **, P < 0.01 vs. control; ***, P < 0.001 vs. control.

Postprandial blood glucose variation was measured after loading sucrose to the normal rats with and without the coadministration of CZ extract. In the control group, blood glucose level increased by an average of 28.6 mg/dl at 30 minutes after the sucrose load. In the group that received CZ extract along with sucrose, the 30 minutes post-load glucose level increased only by 15.8 mg/dl on an average (Figure 7A). This indicates the potency of CZ extract to significantly suppress high sucrose diet associated postprandial glucose elevation. Compared to control, the whole glycemic response is reduced by 42.5% on CZ treatment and 44.6% on acarbose treatment (Figure 7B).

### Sucrose loading in diabetic rats

**Figure 8:**
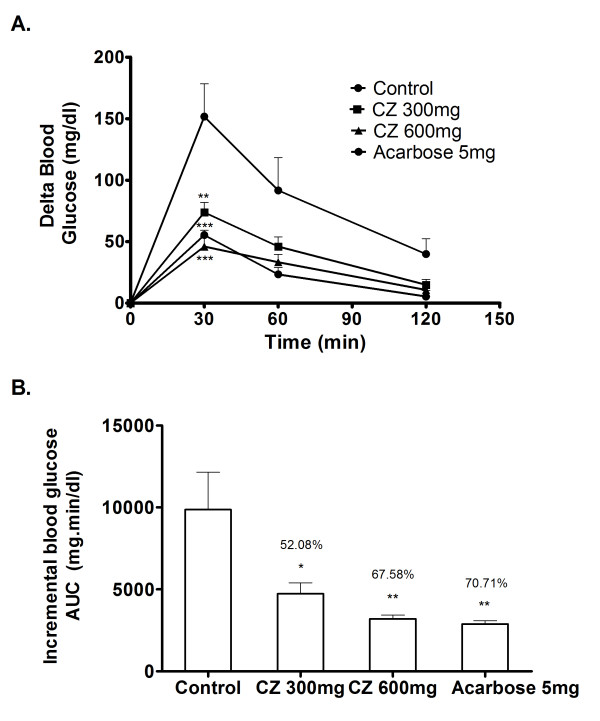
**Inhibitory effect of CZ on blood glucose after sucrose loading in diabetic rats**. The diabetic rats fasted for 16 h received sucrose (2 g/kg body wt; p.o.) and a dose of CZ (300 mg/kg body wt; p.o.) by gastric intubations. Control animals were given only sucrose (2 g/kg body wt; p.o.) and the drug control group received sucrose (2 g/kg body wt; p.o.) plus acarbose (5 mg/kg). Blood glucose was monitored at 0, 30, 60 and 120 min after food administration. A. The glycemic response curve in diabetic rats after sucrose challenge. B. The incremental AUC_0-120 min _in diabetic rats after sucrose administration. Data are expressed as the mean ± S.E, n = 6. *, P < 0.05 vs. control; **, P < 0.01 vs. control; ***, P < 0.001 vs. control.

Postprandial blood glucose variation was measured after loading sucrose to the diabetic rats with and without the coadministration of CZ extract. In the control group, blood glucose level increased to an average of 151.6 mg/dl above the basal level 30 min after CZ administration and decreased thereafter (Figure 8A). However, the rise of the post-load blood glucose has been significantly impeded in a dose dependent fashion on coadministering CZ with sucrose at different doses (300, 600 mg/kg body wt.). Similar kind of suppression effect was observed in the group that received acarbose (5 mg/kg body wt.) as the positive control along with sucrose. Compared to control, the whole glycemic response is reduced by 52.0%, 67.5% and 70.7% when treated with 300, 600 mg/kg body wt of CZ and 5 mg/kg body wt. of acarbose, respectively (Figure 8B).

### Glucose loading in normal rats

**Figure 9:**
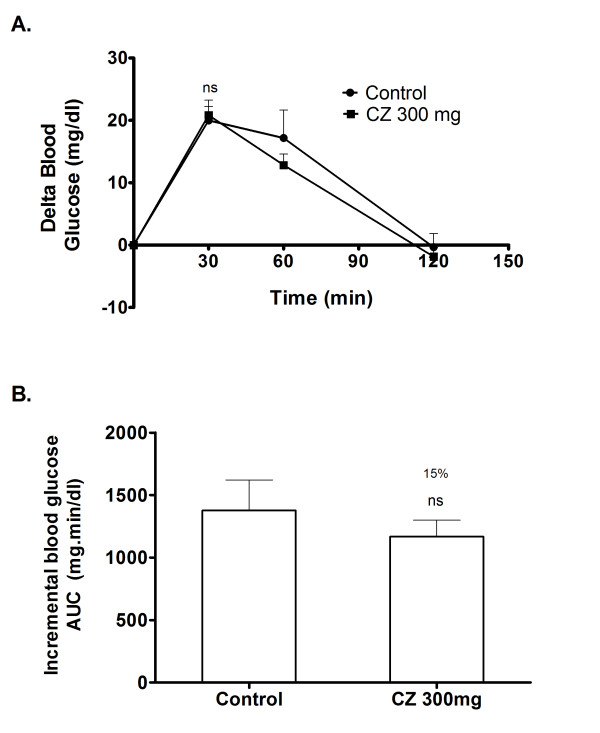
**Inhibitory effect of CZ on blood glucose after glucose loading in normal rats**. The rats fasted for 16 h received glucose (2 g/kg body wt; p.o.) and a dose of CZ (300 mg/kg body wt; p.o.) by gastric intubations. Control animals were given only glucose (2 g/kg body wt; p.o.). Blood glucose was monitored at 0, 30, 60 and 120 min after food administration. There are no significant changes observed in the 30 minutes post-load glucose level between the control group and CZ treated group. Data are expressed as the mean ± S.E, n = 6. ns- not significant.

To affirm that the observed suppression of postprandial glucose is due to the inhibition of α-glucosidase, postprandial blood glucose variation was measured after loading glucose to the normal rats with and without the coadministration of CZ extract. In the control group, blood glucose level increased by an average of 20 mg/dl at 30 minutes after the glucose load. In the group that received CZ extract along with glucose, the 30 minutes post-load glucose level increased by 20.8 mg/dl on an average (Figure 9A), which shows that the glucose absorption is not significantly affected due to CZ extract (Figure 9B).

### Glucose loading in diabetic rats

**Figure 10:**
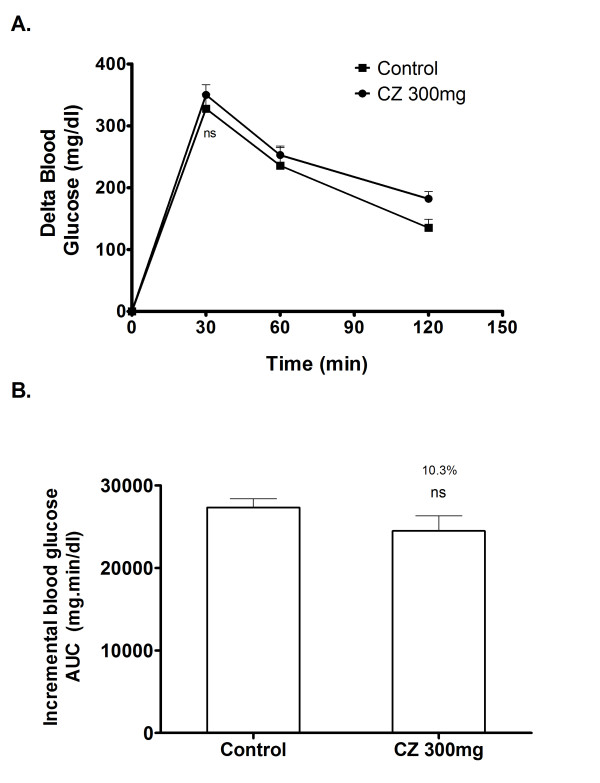
**Inhibitory effect of CZ on blood glucose after glucose loading in diabetic rats**. The diabetic rats fasted for 16 h received glucose (2 g/kg body wt; p.o.) and a dose of CZ (300 mg/kg body wt; p.o.) by gastric intubations. Control animals were given only glucose (2 g/kg body wt; p.o.). Blood glucose was monitored at 0, 30, 60 and 120 min after food administration. There are no significant changes observed in the 30 minutes post-load glucose level between the control group and CZ treated group. Data are expressed as the mean ± S.E, n = 6. ns- not significant.

To evaluate the effect of cinnamon on glucose tolerance in diabetic condition and to elucidate whether the observed postprandial glucose suppression is majorly due to α-glucosidase inhibition, postprandial blood glucose variation was measured after loading glucose to the diabetic rats with and without the coadministration of CZ extract. In the control group, blood glucose level increased by an average of 350.1 mg/dl at 30 minutes after the glucose load. In the group that received CZ extract along with glucose, the 30 minutes post-load glucose level increased by 327.8 mg/dl on an average (Figure 10A), which shows that the glucose absorption is not significantly affected due to CZ extract (Figure 10B).

## Discussion

Diabetic individuals are at an increased risk of developing microvascular complications (retinopathy, nephropathy, and neuropathy) and cardiovascular disease (CVD). Abnormalities in insulin and glucagon secretion, hepatic glucose uptake, suppression of hepatic glucose production, and peripheral glucose uptake contribute to higher and more prolonged postprandial glycemic (PPG) excursions than in non diabetic individuals [2]. Elevated PPG even in the absence of fasting hyperglycemia increases the risk of cardiovascular diseases and it is the most common cause of death among the people with diabetes. Acute hyperglycemia induces endothelial dysfunction by generating oxidative stress resulting in impaired vasodilatation [39]. Also, postprandial spikes can result in microvascular damage through oxidation of low density lipoprotein (LDL) and other proatherogenic mechanisms [40].

Diet rich in carbohydrate causes sharp rise in the blood glucose level as the complex carbohydrates in the food is rapidly absorbed in the intestine aided by the α- glucosidase enzyme which breaks disaccharides into absorbable monosaccharides [41]. α-glucosidase inhibitor inhibits the disaccharide digestion and impedes the postprandial glucose excursion to enable overall smooth glucose profile [42].

The antidiabetic action of cinnamon exerted by insulin secretagogue action and insulin resistance amelioration has been previously reported [17,18]. Ahmad Gholamhoseinian [24] screened 200 Iranian medicinal plants *in vitro *and reported that the cinnamon extract exhibited strong inhibition on yeast α-glucosidase. However, the nature of the enzyme inhibition was not studied. As most of the plant derived inhibitors showing effective inhibition on yeast α-glucosidase do not effectively inhibit the mammalian α-glucosidase, we have prompted to evaluate the effect of cinnamon on mammalian α-glucosidase. In addition, the effect of cinnamon extract on postprandial glucose excursion associated with maltose, sucrose challenge was studied in normal and STZ induced diabetic rats.

The ability of cinnamon bark extract to inhibit the α-glucosidase *in vitro *has been evaluated using yeast α-glucosidase and mammalian α-glucosidase which are commonly used for investigating α-glucosidase inhibitors from microbes and medicinal plants [43]. In our *in vitro *studies, cinnamon extract showed remarkable inhibition on both yeast and mammalian α-glucosidase suggesting the presence of potential enzyme inhibiting compound in the extract. To find the mechanism of inhibition, we have formulated double reciprocal plot from the kinetics data and the results indicate the competitive mode of inhibition of CZ extract similar to acarbose which is also a competitive inhibitor.

In our study, we found that the inhibitory action of cinnamon on α-glucosidase to be reversible: the enzyme activity was recovered intact after dialysis as the process of dialysis cleared the inhibitors from the enzyme. The reversible inhibition is the propitious property of α-glucosidase inhibitor because the enzymes remain intact even after the elimination of the inhibitor. In other words, when inhibitor binds irreversibly to the intestinal enzyme, it will lead to hypoglycemia due to chronic carbohydrate malabsorption.

Following the positive *in vitro *inhibitory results of the cinnamon extract, we have continued to evaluate its effect on postprandial hyperglycemia associated with carbohydrate challenge using rats as our model. The hypothesis is that on administering cinnamon extract to the diabetic rats, postprandial glucose excursion associated maltose or sucrose challenge gets stymied but not during glucose challenge. Because, the α-glucosidase action is crucial for the digestion of maltose and sucrose without which these disaccharides would not be rapidly converted into absorbable glucose. As expected, cinnamon extract blunted acute postprandial hyperglycemic spike in the normal wistar rats loaded with maltose and sucrose but not with glucose. Subsequently, the postprandial hyperglycemia amelioration of cinnamon extract was evaluated in the STZ induced diabetic wistar rats. In general, the postprandial glucose level of STZ induced diabetic rat is poorly controlled due to impaired insulin production [44]. However, in our study, coadministration of maltose or sucrose along with cinnamon extract to the diabetic rats prevented the sharp hike in a dose dependent manner. On the other hand, control animals showed an extremely high level of blood glucose that has been staying high even two hours after the maltose or sucrose load. One of the reasons for observing the suppressed postprandial glucose level in diabetic rats could be due to the damping effect of cinnamon extract on the maltose or sucrose digestion at small intestine. The standard drug, acarbose similarly suppressed the postprandial glucose level. As the observed postprandial glucose suppression could also be possible because of the secretagogue activity and insulin sensitizing property of cinnamon, we have evaluated the effect of cinnamon on glucose loading in the normal and diabetic rats. Cinnamon did not suppress the postprandial hyperglycemia associated with glucose (monosaccharide) loading significantly but on maltose and sucrose (disaccharide) loading, which shows that the major mechanism of action of postprandial glucose suppression is exhibited by inhibition of α-glucosidase. To precisely understand the mechanism of enzyme inhibition, we are on the process of purifying and isolating an active compound(s) and determine its chemical structure for further study.

The phytochemical analysis indicated the presence of flavonoids and glycosides along with other major common secondary metabolites in the extract. Previous reports on α-glucosidase inhibitors isolated from medicinal plants suggest that several potential inhibitors belong to flavonoid glycoside class which has the characteristic structural features to inhibit α-glucosidase enzyme [45] - [46]. Based on the preliminary results obtained from our LC-MS study (data not shown), we speculate that the presence of flavonoid glycosides might have contributed to the α-glucosidase inhibitory effect of the cinnamon extract.

## Conclusions

Cinnamon bark extract shows competitive, reversible inhibition on α-glucosidase enzyme. It effectively suppresses the maltose and sucrose induced postprandial blood glucose spikes in rats. Cinnamon extract could be used as a potential nutraceutical agent for treating postprandial hyperglycemia. In future, specific inhibitor has to be isolated from the crude extract, characterized and therapeutically exploited.

## List of abbreviations

AGI: α-glucosidase inhibitor; CZ: *Cinnamomum zeylanicum*; STZ: Streptozotocin; PPG: Postprandial glucose; PPHG: Postprandial hyperglycemia; IC: Inhibitory concentration; LB plot: Lineweaver-Burk plot; PNPG: *p*-Nitrophenyl-α-D-glucopyranoside; CVD: Cardiovascular diseases.

## Competing interests

The authors declare that they have no competing interests.

## Authors' contributions

MSS carried out the *in vitro *and *in vivo *experiments, participated in its design, analysed and interpreted the data and drafted the manuscript. KT conceived of the study, designed, coordinated, involved in drafting the manuscript and revised it critically. HPD participated in the *in vivo *studies and helped drafting the manuscript.

All authors read and approved the final manuscript.
